# Comparison of Productivity and Fecal Microbiotas of Sows in Commercial Farms

**DOI:** 10.3390/microorganisms8101469

**Published:** 2020-09-24

**Authors:** Haruka Uryu, Takamitsu Tsukahara, Hiromichi Ishikawa, Munetaka Oi, Satoshi Otake, Itsuro Yamane, Ryo Inoue

**Affiliations:** 1 Laboratory of Animal Science, Division of Applied Life Sciences, Graduate School of Life and Environmental Sciences, Kyoto Prefectural University, Kyoto 606-8522, Japan; s819631006@kpu.ac.jp; 2 Laboratory of Animal Science, Department of Applied Biological Sciences, Faculty of Agriculture, Setsunan University, Hirakata, Osaka 573-0101, Japan; 3 Kyoto Institute of Nutrition & Pathology, Kyoto 610-0231, Japan; tsukahara@kyoto-inp.co.jp; 4 The Japanese Association of Swine Veterinarians (JASV), Ibaraki 300-1260, Japan; hiromichi.ishikawa.jasv@gmail.com (H.I.); munetaka.oi.jasv@gmail.com (M.O.); satoshi.otake.jasv@gmail.com (S.O.); 5 National Agriculture and Food Research Organization (NARO) (National Institute of Animal Health), Ibaraki 305-0856, Japan; iyamane@affrc.go.jp

**Keywords:** sow productivity, fecal microbiota, gut microbiota, short-chain fatty acid, fiber degrading, farm

## Abstract

Sow productivity, that is, the number of weaned piglets per sow per year, depends on their health status. The gut microbiota is considered a crucial factor in the health of pigs and may affect sow productivity. In the present study, we aimed to investigate the relationship between productivity and the fecal microbiotas of sows in different farms. Feces of sows were collected from 18 farms (10 samples/farm). A total of 90 fecal samples of high-reproductive performance farms were labeled as group H, and 90 fecal samples from low-reproductive performance farms were labeled as group L. Fecal microbiotas were analyzed by 16S rRNA metagenomics, and the organic acids and putrefactive metabolites of the microbiotas were measured. β-diversity was significantly different between groups H and L (*p <* 0.01), and the relative abundances of 43 bacterial genera, including short-chain fatty acid-producing and fiber-degrading bacteria such as *Ruminococcus*, *Fibrobacter* and *Butyricicoccus*, significantly differed between groups (*p <* 0.05). In addition, the concentrations of acetate, propionate and *n*-butyrate were significantly higher in group H than in group L (*p* < 0.05). In conclusion, sow productivity in farms was likely associated with the compositions of the fecal microbiotas.

## 1. Introduction

The number of piglets produced per sow in a year depends on the sow’s reproductive performance [1]. In addition, the number of weaned piglets per sow in a year, a major productivity parameter, is defined by several factors such as the number of piglets born alive, the number of surviving piglets during suckling, as well as the days of recurrence to estrus and the pregnancy rate of the sow [1]. Therefore, “the number of weaned pigs/sow/year” is also considered a major parameter to evaluate farm productivity [2]. During lactation, the loss of body condition in sows induces a delay in the recurrence to estrus, and subsequent decreases in the pregnancy rate and the litter size [3]. Moreover, increases in stress in prepartum sows cause lower maternal endogenous hormone levels and negatively affect the farrowing performance, mostly due to prolonged birth intervals [4]. Therefore, the health conditions of sows directly affect their reproductive performance.

The gut microbiotas are the bacterial populations that reside in the intestinal tract of the host. The number of bacteria in the gut microbiotas of mammals is estimated to be approximately 10^14^ [5,6], which maintain a relatively stable crosstalk with their complex ecosystem. The gut microbiota exerts certain beneficial effects on the host. For example, bacteria in the gut microbiota produce beneficial metabolites such as short-chain fatty acids (SCFAs) that control the proliferation and differentiation of epithelial cells, develop and maintain the homoeostasis of the immune system, and protect the host from pathogens [7,8]. Nonetheless, given the opportunity, some bacteria in the microbiota and their metabolites can adversely affect the host [9,10,11]. In other words, the bacteria in the gut microbiota can be both beneficial and harmful to the host.

The gut microbiota of the pig can also be regarded as a crucial factor in the pig’s health, as it affects the function of the intestinal barrier, the development of the immune system, and the efficiency by which feed is used [12]. Previous work has shown that growing pigs with high-feed efficiency exhibit specific bacterial compositions of their gut microbiotas [13,14,15]. This evidence seems to suggest that the gut microbiotas in growing pigs are associated with their growth performance by promoting healthy intestinal environments. In addition, previous studies showed that status of the gut microbiotas of sows is associated with their reproductive performance [16,17]. However, those studies were carried out in one single farm, focusing solely on finding differences in the productive performance of individual pigs, not between pig farms.

In general, pigs in commercial farms are not managed individually, but per pen, house or farm [18]. Farmers and veterinarians only act and respond when an adverse event in a given pig unit occurs to prevent pathogen infections or changes in the feeding strategy, water quality and stress care [19]. Therefore, we theorized that the indigenous gut microbiotas of sows within a farm would be similar, and hence, more closely associated with sow productivity within the respective farm than with individual pigs.

In the present study, we aimed to investigate the relationship between sow productivity and the fecal microbiota of pigs in different farms. We also investigated the relation between sow productivity and the metabolites in the gut microbiota such as SCFAs, phenol, indole, skatole and para-cresol, because we also theorized that these metabolites may affect the health of sows. To the best of our knowledge, this is the first work to study the relationship between the fecal microbiotas of sows and sow productivity at a farm level. For practical purposes, in the present study, the number of pigs produced per sow/year, that is, the breeding ability of sows, was defined as “sow productivity”.

## 2. Materials and Methods

### 2.1. Animals and Sampling

Pigs from 18 commercial swine farms were used in the present study. These farms were included in the database survey of PigINFO 2017. PigINFO is a benchmarking system operated by the Japanese Association of Swine Veterinarians (JASV) and the Agri-Food Business Innovation Center of National Agriculture and Food Research Organization [20]. In 2017, 162 farms were included in the database. Of all farms, 18 farms were selected, as per breeding lines (L × W or W × L) and reproductive performance (the mean number of piglets weaned per sow/year). Nine farms were from the top tier (top 25% of farms) and 9 from the lowest tier (bottom 25%) of the reproductive performance range. We obtained the consent and fecal samples of 10 sows/farm from the owners of the 18 farms. All experimental procedures were followed in accordance with the guidelines issued by the Ministry of Education, Culture, Sports, Science and Technology-Japan and Science Council of Japan. The experiments were exempted from ethic evaluations because all animals used were commercially raised and reared in a conventional swine farms under the supervision of the local veterinary and the samples were collected on site. Fecal samples were collected fresh from 10 healthy multiparous sows (2nd–5th parity) with fresh feces and used as the fecal samples. Feces during or immediately after defecation was collected by a sterilized fecal collection tube (80.623; SARSTEDT, Tokyo, Japan). When feces fell down onto the ground, the portion that did not touch the ground was carefully chosen and collected. The fecal samples were stored at −80 °C until further use. Ninety fecal samples from the high-reproductive performing farms were labeled as the high-performance group (group H), and the remaining 90 fecal samples were labeled as the low-performance group (group L). The seven reproductive performances parameters used in the present study are shown in Table 1.

### 2.2. Analysis of the Fecal Microbiota by 16S rRNA Metagenomics

Extraction of bacterial DNA from fecal samples, library preparation and deep sequencing by MiSeq (Illumina, Tokyo, Japan) were carried out exactly as described by Inoue et al. [21]. The obtained sequence data were analyzed as per the protocol reported by Inoue et al. [21].

### 2.3. Measurement of the Concentrations of Organic Acids in Fecal Samples

The number of samples with measurable organic acid concentrations were 87 in group H and 88 in group L, because the volumes of three samples in group H and two in group L did not suffice for measurement.

The concentrations of fecal organic acids such as succinate, lactate, formate, acetate, propionate, iso-butyrate, *n*-butyrate, iso-valerate and *n*-valerate were measured by ion-exclusion, high-performance liquid chromatography. The procedure was the same as that described elsewhere [22].

### 2.4. Measurement of the Concentrations of Putrefactive Metabolites in Fecal Samples

Similar to those for organic acid measurement, the number of samples with measurable putrefactive metabolite concentrations were 76 in group H and 87 in group L, because the volumes of 14 samples in group H and three in group L did not suffice for measurement.

The concentrations of fecal putrefactive metabolites such as phenol, indole, skatole, para-ethylphenol and para-cresol were measured by gas chromatography-mass spectrometry. The procedure was the same as that described elsewhere [23].

### 2.5. Statistical Analysis

The differences in the parameters of reproductive performances between groups H and L were analyzed by the Welch’s *t*-test, using Excel software statcel2 (OMS Publishing, Saitama, Japan). In addition, the number of piglets weaned per sow/year were correlated with other reproductive performances by the Spearman’s rank correlation test, using Excel software statcel2.

Alpha-(α-) diversity Chao1 (richness) and Shannon (evenness) indices were calculated with the R phyloseq package. Chao1 and Shannon indices between groups H and L were statistically compared with Mann–Whitney’s U-test, using Excel software statcel2.

Beta-(β-)diversity was estimated using UniFrac distances and tested by a principal coordinate analysis (PCoA), using the R phyloseq package. The UniFrac distance between groups H and L was statistically analyzed by permutational multivariate analysis of variance (PERMANOVA), using QIIME1.9.1. The relative abundances (%) of all detected bacterial genera between groups H and L were calculated and statistically compared with the Welch’s *t*-test, and adjusted for the false discovery rate with the Storey’s method using STAMP software (statistical analysis of taxonomic and functional profiles) [24].

The concentrations of organic acids and putrefactive metabolites between groups H and L were statistically compared with the Wilcoxon rank sum test, using R 4.0.0.

Chao1 and Shannon indices between 18 farms were also analyzed by one-way analysis of variance (ANOVA), using Excel software statcel2. The UniFrac distances between 18 farms and between 9 farms in each group were statistically analyzed by permutational multivariate analysis of variance (PERMANOVA), using QIIME1.9.1.

Differences between the means were considered significant if *p* < 0.05 and, in the case of the relative abundances of bacterial genera, if *p* < 0.05 and *q* < 0.1. Values are expressed as the means ± the standard errors.

## 3. Results

### 3.1. Reproductive Parameters

Five parameters of reproductive performance, including farrowing rate (farrowing of inseminated sows), were significantly different between groups (*p <* 0.01, Table 1). However, neither the number of stillbirths per litter nor pre-weaning mortality were significantly different between groups.

When correlations between the number of pigs weaned per sow/year and six parameters of reproductive performance were analyzed, four parameters showed positive correlations (*p <* 0.01, Table 2). Nonetheless, neither the number of stillbirths per litter nor pre-weaning mortality were correlated with the number of piglets weaned per sow/year.

### 3.2. Comparison of the Fecal Microbiotas of High- and Low-Reproductive Performance Groups

**Figure 1:**
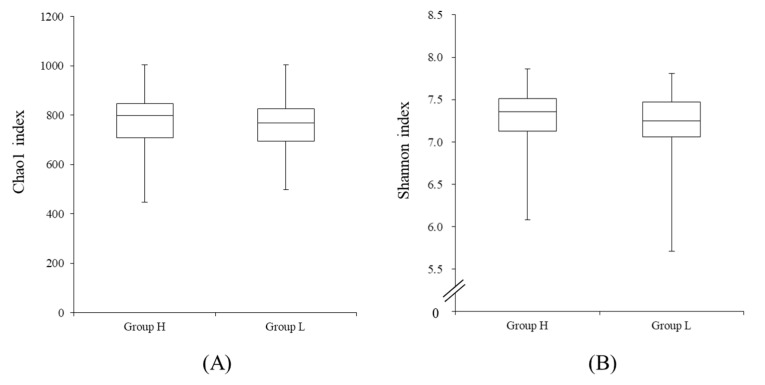
Chao1 (**A**) and Shannon (**B**) indices of the fecal microbiotas in high- and low-reproductive performance groups. Panel A: Chao1 index; Panel B: Shannon index.

**Figure 2:**
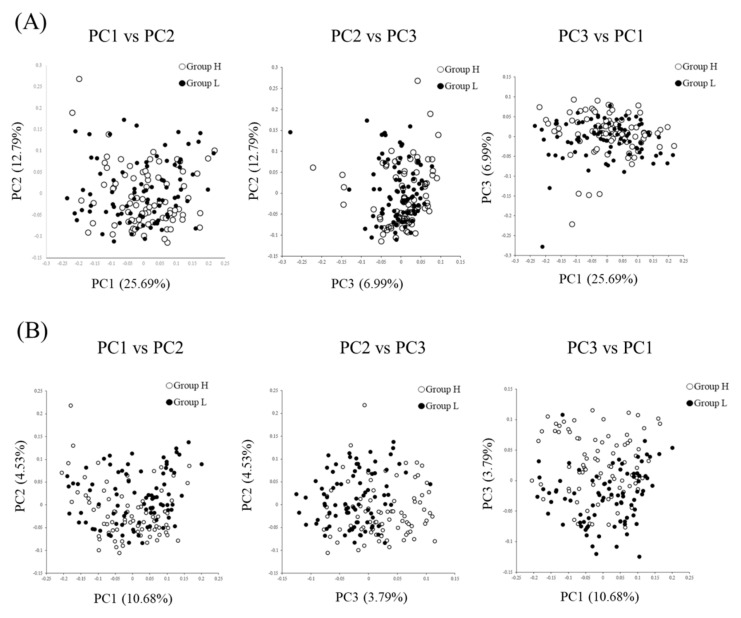
Principal coordinate analysis (PCoA) based on weighted UniFrac (**A**) and unweighted UniFrac (**B**) distances generated from the abundances of the fecal microbiotas. The PERMANOVA analysis indicated that the composition of the fecal microbiotas differed between groups H and L (*p* < 0.01).

The sequence data have been deposited in the DDBJ Sequence Read Archive (DRA) under accession number DRA010825 (available from 1/Nov/2020). α-diversity of both Chao1 (richness) and Shannon (evenness) indices did not significantly differ between groups (Figure 1). β-diversity was significantly different between groups (*p <* 0.01, Figure 2). The relative abundances of 43 bacterial genera were significantly different between groups (*p <* 0.05, *q* < 0.1, Table 3). Among genera of which taxonomy was classified and detected to be higher than 0.1%, *Treponema*, *Ruminococcus*, *Collinsella*, *Fibrobacter*, *Phascolarctobacterium*, *Rummeliibacillus*, *Butyricicoccus*, *Bulleidia*, *Oribacterium*, *Blautia*, *Sphaerochaeta* and *Peptococcus,* were detected to be more abundant in group H than in group L. By contrast, genera *Streptococcus*, *CF231*, *Parabacteroides* and *Campylobacter* were found to be more abundant in group L than in group H.

### 3.3. Metabolites of the Microbiota in Feces

The concentrations of microbial metabolites in the fecal samples of sows are shown in Table 4. The concentrations of acetate, propionate and *n*-butyrate were significantly higher in group H than in group L (*p* < 0.05). However, the concentrations other organic acids did not significantly differ between groups

The concentrations of putrefactive metabolites are shown in Table 5. No significant differences in putrefactive metabolites were detected between groups.

## 4. Discussion

To maintain a stable management of swine farms, the reproductive performance of sows needs to be taken into consideration. Recently, Shao et al. [16] suggested that the microbial populations in the gut were clearly affected by the reproductive performance of sows in a single farm. Shao et al. [16] divided their sows into high-reproductive and low-reproductive groups according to their litter sizes, while in the present work, we used the parameter “number of weaned pigs per sow per year” to define sow productivity and hence divide the farms. We confirmed this parameter properly correlated with the other reproductive parameters (*p <* 0.05, Table 2), and therefore, was robust enough to evaluate the reproductive performance of sows in a farm.

In the present work, the fecal microbiotas of sows markedly differed between the high- and low-reproductive performance farms. While differences were clearly obtained by the β-diversity analysis (Figure 2), based on both unweighted and weighted UniFrac distances, no differences were found for the α-diversity in the experimental groups (Figure 1). According to Shao et al. [16], not only β-diversity but also α-diversity differed between high- and low-performance sows. Therefore, there seemed to be a discrepancy between the α-diversity index in the present study and that of Shao et al. [16]. When we compared the α-diversity index of the farms studied in the present work, Chao1 and Shannon indices differed, regardless of the reproductive performance (Figure S1). Hence, we believe that the comparison of α-diversity indices may be inadequate if the gut microbiotas are linked to the reproductive performance of sows in farms. Nonetheless, in the present study, the β-diversity index was clearly different between high- and low-performance groups, whether a single farm [16] or several farms were studied. Therefore, we concluded that the β-diversity, not α-diversity, is a crucial factor to evaluate the effect of gut microbiotas on the reproductive performance of sows.

According to the relative abundance at the genus level (Table 3), 43 bacterial genera (*p <* 0.05) were significantly different between the high- and low-performance groups. When observing the taxonomically classified genera whose abundances were higher than 0.1%, the abundances of *Treponema*, *Ruminococcus*, *Collinsella*, *Fibrobacter*, *Phascolarctobacterium*, *Rummeliibacillus*, *Butyricicoccus*, *Bulleidia*, *Oribacterium*, *Blautia*, *Sphaerochaeta* and *Peptococcus* were higher in group H than in group L. Shao et al. [16] reported that the feces of high-reproductive performance sows contained high abundances of genera *Colinsella*, *Fibrobacter*, *Phascolarctobacterium*, and *Sphaerochaeta*, which align with the results from the present work. In addition, most of the genera with high relative abundances in group H were SCFA-producing and/or fiber-degrading bacteria, which have been associated with high feed efficiency and good health of pigs. For example, *Butyricicoccus*, *Blautia*, *Bulleidia* and *Oribacterium* are known to produce beneficial SCFAs (acetate and *n*-butyrate) which putatively contribute to the health of pigs [25,26,27,28]. Separately, *Fibrobacter* and *Ruminococcus* are known to degrade dietary fiber and produce SCFAs [29,30], and have been found to be abundant in the feces of sows fed a diet high in crude fiber and to improve the farrowing performance of sows [31]. The concentrations of acetate, propionate and n-butyrate were significantly higher in group H than in group L (*p <* 0.05), which further substantiate the aforementioned evidence of the presence of high SCFA-producing microbiota in group H.

SCFA produced in the large intestine are rapidly absorbed from the mucosa and used as metabolic energy [8,32]. Furthermore, SCFAs, *n*-butyrate in particular, support major functions of the epithelial cells, such as water and mineral absorption [33] and mucus production [34] in the large intestine. This energy supply from the large intestine may well contribute to the health of sows and subsequently help improve their reproductivity. SCFAs also contribute to the regulation of the mucosal immunity, including the production of regulatory T cells [35]. A recent report suggested that acetate produced in the large intestine may exert an anxiolytic effect on the host [36], which may have contributed to better reproductivity of sows in group H.

In contrast, group L had only four genera, namely *Streptococcus*, *CF231*, *Parabacteroides* and *Campylobacter*, whose abundances were higher than in group H (Table 3). It has been reported that the abundance of *Streptococcus* spp. is low in the feces of pigs with high-feed efficiency [15]. Similarly, Shao et al. [16] also reported that the abundance of *Streptococcus* was low in sows with high reproductive performance. *Streptococcus suis* is a major swine pathogen that causes a decrease in performance and an increase in mortality [37,38]. However, detailed analysis of OTUs (operational taxonomy units) belonging to genera *Streptococcus* suggested that abundance of *Streptococcus luteciae* was different between groups H and L. The role of *S. luteciae* in the swine industry is currently not clear but it is possible that *S. luteciae* affects the health of sows. Genus *CF231* belonging to the phylum *Bacteroidetes* is high in the colonic digesta of pigs with high feed efficiency, but the biological functions of *CF231* remain unclear as it is yet to be cultured in vitro [39]. Genus *Parabacteroides* is abundant in the feces of growing pigs with low body weights, and Oh et al. [40] reported that *Parabacteroides* had a negative correlation with body weight and average daily gain. Lin et al. [41] also reported that reduction of *Parabacteroides* in the colon may help enhance the health of the intestine. Genus *Campylobacter* spp. is known to play a role in the aggravation of diarrhea and thus, is relatively abundant in piglets suffering from diarrhea [42,43]. In addition, De Rodas et al. [44] reported that *Campylobacter* was negatively correlated with body weight in growing pigs. Therefore, a high abundance of these four potentially harmful genera may adversely affect the productivity of sows.

No differences were found in the concentrations of putrefactive metabolites in feces between sow groups (Table 5). Putrefactive metabolites are produced by intestinal bacteria that degrade proteins, and a part of these metabolites enter the bloodstream from the large intestine [45]. Thus, we theorized that given the lack of differences, putrefactive metabolites were not deeply associated with the reproductivity of sows.

It is worth noting that the compositions of the gut microbiotas seemed to be unique in sows of each farm. α-diversity of both Chao1 and Shannon indices significantly differed between the 18 farms (*p* < 0.01, Figure S1). UniFrac distances of the microbiotas in the feces between the 18 farms were also significantly different (*p <* 0.01). When we analyzed the UniFrac distance between nine farms in either group H or L, the fecal microbiotas were also significantly different (*p <* 0.01, Figure S2). Moreover, UniFac distance (both weighted and unweighted) of fecal microbiotas in sows within same farm (intra-farm distance) was significantly lower than the distance against fecal microbiota of sows in different farms (inter-farm distance) in most of the farms (Figure S3). The gut microbiota is thought to exist within a relatively stable ecosystem, but this may be changed by pathogens, drugs, food and stress [46]. In addition, the gut microbiota of pigs in farms may be changed by factors of the surrounding environment such as temperature, overcrowded areas and housing system [47,48]. In a recent report, it has also been suggested that these factors can affect the gut microbiota of sows even between farms of the same production company and contiguous locations [49]. Our results seem to be in agreement with the existing evidence, as we showed that the indigenous fecal microbiota differed not only between individual pigs but also between farms.

Because the fecal samples were collected from commercial farms, the diet (feed) of sows is not normalized in this study. This might be considered as a limiting factor of this study as the difference in feed could affect the composition of fecal microbiota. However, we have confirmed sows in all farms were given at least a complete feed mixture and most of the farms used commercially available feed. Additionally, one farm in H group and two farms in L group used feed from same company. Therefore, we believe difference in feed may not a decisive factor of difference in fecal microbiota observed between H and L groups, although it should contribute at least partly to the uniqueness of fecal microbiota in each farm.

## 5. Conclusions

In conclusion, our results suggested that the compositions of the fecal microbiotas were associated with sow productivity even at a farm level. The PCoA analysis showed that the fecal microbiotas were markedly different between high- and low-reproductive performance farms. Finally, SCFA-producing and/or fiber-degrading bacteria and accordingly, fecal SCFAs, were detected more abundantly in high-reproductive performance farms.

## Figures and Tables

**Table 1:** Parameters of reproductive performance in high- and low-reproductive performance groups.

Parameters of Reproductive Performance	Group H (*n* = 9)	Group L (*n* = 9)	Welch’s *t*-Test *p* Values
**Number of piglets weaned/sow/year**	**27.69 ± 0.33**	**19.33 ± 0.62**	**5.3 × 10^−8^**
**Farrowing rate**	**0.89 ± 0.01**	**0.80 ± 0.02**	**2.7 × 10^−3^**
**Farrowing/sow/year**	**2.41 ± 0.02**	**2.21 ± 0.02**	**4.3 × 10^−6^**
**Number of piglets born/litter**	**14.68 ± 0.43**	**11.48 ± 0.30**	**2.4 × 10^−5^**
**Number of piglets born alive/litter**	**13.27 ± 0.33**	**10.24 ± 0.32**	**6.9 × 10^−6^**
Number of stillbirths/litters	1.42 ± 0.14	1.23 ± 0.17	0.42
Pre-weaning mortality	0.13 ± 0.02	0.15 ± 0.02	0.53

Farrowing rate: Farrowing of inseminated sows. Parameters with significant differences between groups H and L are shown in bold (*p* < 0.05). Mean ± SE is shown.

**Table 2:** Correlation analysis of the number of piglets weaned per sow per year and six reproductive performance parameters.

Parameters of Reproductive Performance	Correlation with the Number of Piglets Weaned Per Sow Per Year	
	Correlation Rate	*p*-Value
**Farrowing rate**	**0.71**	**3.37 × 10^−3^**
**Farrowing/sow/year**	**0.75**	**2.04 × 10^−3^**
**Number of piglets born/litter**	**0.88**	**2.93 × 10^−4^**
**Number of piglets born alive/litter**	**0.88**	**2.75 × 10^−4^**
Number of stillbirths/litter	0.18	0.47
Pre-weaning mortality	−0.18	0.45

Parameters with significant correlation between groups H and L are shown in bold (*p* < 0.05).

**Table 3:** Relative abundances of 43 bacterial genera that significantly differed between high- and low-reproductive performance groups.

Phylum	Class	Order	Family	Genus	Group H	Group L
Firmicutes	Clostridia	Clostridiales	Unclassified	Unclassified	5.81 ± 0.25	6.79 ± 0.33
**Spirochaetes**	**Spirochaetes**	**Spirochaetales**	***Spirochaetaceae***	***Treponema***	**4.79 ± 0.31**	**3.59 ± 0.24**
**Firmicutes**	**Clostridia**	**Clostridiales**	***Ruminococcaceae***	***Ruminococcus***	**3.76 ± 0.19**	**3.10 ± 0.16**
Planctomycetes	Planctomycetia	Pirellulales	*Pirellulaceae*	Unclassified	1.92 ± 0.17	3.01 ± 0.35
Verrucomicrobia	Verruco-5	WCHB1-41	*RFP12*	Unclassified	0.87 ± 0.07	0.62 ± 0.05
Actinobacteria	Coriobacteriia	Coriobacteriales	*Coriobacteriaceae*	Unclassified	0.76 ± 0.04	0.62 ± 0.03
**Firmicutes**	**Bacilli**	**Lactobacillales**	***Streptococcaceae***	***Streptococcus***	**0.68 ± 0.14**	**1.99 ± 0.28**
**Fibrobacteres**	**Fibrobacteria**	**Fibrobacterales**	***Fibrobacteraceae***	***Fibrobacter***	**0.57 ± 0.07**	**0.37 ± 0.04**
**Bacteroidetes**	**Bacteroidia**	**Bacteroidales**	**[*Paraprevotellaceae*]**	***CF231***	**0.54 ± 0.05**	**0.73 ± 0.07**
**Bacteroidetes**	**Bacteroidia**	**Bacteroidales**	***Porphyromonadaceae***	***Parabacteroides***	**0.50 ± 0.04**	**0.67 ± 0.04**
**Firmicutes**	**Bacilli**	**Bacillales**	***Planococcaceae***	***Rummeliibacillus***	**0.49 ± 0.18**	**0.03 ± 0.01**
**Firmicutes**	**Clostridia**	**Clostridiales**	***Veillonellaceae***	***Phascolarctobacterium***	**0.49 ± 0.03**	**0.36 ± 0.02**
**Firmicutes**	**Clostridia**	**Clostridiales**	***Ruminococcaceae***	***Butyricicoccus***	**0.44 ± 0.03**	**0.30 ± 0.03**
**Firmicutes**	**Erysipelotrichi**	**Erysipelotrichales**	***Erysipelotrichaceae***	***Bulleidia***	**0.39 ± 0.04**	**0.24 ± 0.02**
**Firmicutes**	**Clostridia**	**Clostridiales**	***Lachnospiraceae***	***Oribacterium***	**0.32 ± 0.05**	**0.16 ± 0.03**
**Firmicutes**	**Clostridia**	**Clostridiales**	***Lachnospiraceae***	***Blautia***	**0.27 ± 0.03**	**0.17 ± 0.01**
**Spirochaetes**	**Spirochaetes**	**Sphaerochaetales**	***Sphaerochaetaceae***	***Sphaerochaeta***	**0.19 ± 0.01**	**0.10 ± 0.01**
Bacteroidetes	Bacteroidia	Bacteroidales	*RF16*	Unclassified	0.18 ± 0.02	0.08 ± 0.01
**Actinobacteria**	**Coriobacteriia**	**Coriobacteriales**	***Coriobacteriaceae***	***Collinsella***	**0.17 ± 0.02**	**0.06 ± 0.01**
**Firmicutes**	**Clostridia**	**Clostridiales**	***Peptococcaceae***	***Peptococcus***	**0.13 ± 0.01**	**0.10 ± 0.01**
**Proteobacteria**	**Epsilonproteobacteria**	**Campylobacterales**	***Campylobacteraceae***	***Campylobacter***	**0.08 ± 0.01**	**0.16 ± 0.04**
**Proteobacteria**	**Betaproteobacteria**	**Burkholderiales**	***Oxalobacteraceae***	***Oxalobacter***	**0.07 ± 0.01**	**0.03 ± 0.00**
Lentisphaerae	[Lentisphaeria]	Z20	*R4-45B*	Unclassified	0.07 ± 0.01	0.02 ± 0.00
**Tenericutes**	**Mollicutes**	**Anaeroplasmatales**	***Anaeroplasmataceae***	***Anaeroplasma***	**0.05 ± 0.01**	**0.03 ± 0.00**
Verrucomicrobia	Verruco-5	WCHB1-41	Unclassified	Unclassified	0.05 ± 0.01	0.02 ± 0.00
**Firmicutes**	**Erysipelotrichi**	**Erysipelotrichales**	***Erysipelotrichaceae***	***RFN20***	**0.04 ± 0.00**	**0.03 ± 0.00**
**Synergistetes**	**Synergistia**	**Synergistales**	***Synergistaceae***	***Synergistes***	**0.03 ± 0.01**	**0.01 ± 0.00**
**Firmicutes**	**Clostridia**	**Clostridiales**	***Lachnospiraceae***	***Lachnospira***	**0.02 ± 0.01**	**0.01 ± 0.00**
**Firmicutes**	**Bacilli**	**Lactobacillales**	***Enterococcaceae***	***Enterococcus***	**0.02 ± 0.01**	**0.01 ± 0.00**
**Firmicutes**	**Clostridia**	**Clostridiales**	***Eubacteriaceae***	***Anaerofustis***	**0.02 ± 0.00**	**0.02 ± 0.00**
Verrucomicrobia	Verruco-5	WCHB1-41	*WCHB1-25*	Unclassified	0.02 ± 0.00	0.01 ± 0.00
Proteobacteria	Alphaproteobacteria	RF32	Unclassified	Unclassified	0.02 ± 0.00	0.01 ± 0.00
Tenericutes	RF3	ML615J-28	Unclassified	Unclassified	0.01 ± 0.00	0.01 ± 0.00
Elusimicrobia	Elusimicrobia	Elusimicrobiales	*Elusimicrobiaceae*	Unclassified	0.01 ± 0.00	<0.01
Lentisphaerae	[Lentisphaeria]	Victivallales	*Victivallaceae*	Unclassified	0.01 ± 0.00	<0.01
**Firmicutes**	**Bacilli**	**Lactobacillales**	***Leuconostocaceae***	***Weissella***	**0.01 ± 0.00**	**0.03 ± 0.01**
**Firmicutes**	**Erysipelotrichi**	**Erysipelotrichales**	***Erysipelotrichaceae***	***Asteroleplasma***	**0.01 ± 0.00**	**<0.01**
Bacteroidetes	Bacteroidia	Bacteroidales	*Rikenellaceae*	Unclassified	<0.01	<0.01
**Firmicutes**	**Clostridia**	**Clostridiales**	***Ruminococcaceae***	***Anaerofilum***	**<0.01**	**0.02 ± 0.00**
**Deferribacteres**	**Deferribacteres**	**Deferribacterales**	***Deferribacteraceae***	***Mucispirillum***	**<0.01**	**0.01 ± 0.00**
**Firmicutes**	**Bacilli**	**Lactobacillales**	***Leuconostocaceae***	***Leuconostoc***	**<0.01**	**0.02 ± 0.01**
**Fusobacteria**	**Fusobacteriia**	**Fusobacteriales**	***Fusobacteriaceae***	***Fusobacterium***	**<0.01**	**0.06 ± 0.03**
**Firmicutes**	**Clostridia**	**Clostridiales**	**[*Tissierellaceae*]**	***Parvimonas***	**<0.01**	**<0.01**

The relative abundances of 43 bacterial genera were significantly different between groups H and L (*p <* 0.05, *q* < 0.1). Thirty bacterial genera whose names were identified are shown in bold. Mean ± SE is shown.

**Table 4:** Organic acid concentrations in fecal samples of high- and low-reproductive performance groups.

Organic Acids (µmol/g of Wet Feces)	Group H (*n* = 87)	Group L (*n* = 88)	Wilcoxon Rank Sum Test *p* Values
Lactate	0.13 ± 0.05	0.24 ± 0.05	0.20
Formate	0.02 ± 0.02	0.01 ± 0.01	0.99
**Acetate**	**86.53 ± 2.43**	**81.00 ± 2.73**	**0.04**
**Propionate**	**37.49 ± 1.27**	**33.28 ± 1.61**	**0.01**
iso-Butyrate	0.13 ± 0.14	0.24 ± 0.16	0.36
**n-Butyrate**	**16.11 ± 0.79**	**14.48 ± 0.96**	**0.045**
iso-Valerate	0.13 ± 0.22	0.24 ± 0.24	0.38
n-Valerate	0.13 ± 0.17	0.24 ± 0.21	0.93

No sample had a detectable concentration of succinate. The concentrations of organic acids that significant differed between groups H and L are shown in bold letters (*p* < 0.05). Mean ± SE is shown.

**Table 5:** Putrefactive metabolites concentrations in fecal samples of high- and low-reproductive performance groups.

Putrefactive Metabolites (µmol/g of Wet Feces)	Group H (*n* = 76)	Group L (*n* = 87)	Wilcoxon Rank Sum Test *p* Values
phenol	52.67 ± 4.34	51.56 ± 2.79	0.77
para-cresol	62.95 ± 5.08	61.32 ± 3.32	0.81
indole	2.32 ± 0.21	2.30 ± 0.19	0.95
para-ethylphenol	0.19 ± 0.03	0.18 ± 0.03	0.68
skatole	9.54 ± 0.88	10.97 ± 0.81	0.13

No significant differences in the concentrations of putrefactive metabolites were observed between groups H and L (*p* > 0.1). Mean ± SE is shown.

## Supplementary Materials

The following are available online at https://www.mdpi.com/2076-2607/8/10/1469/s1. Figure S1: Chao1 (A) and Shannon (B) indices of the fecal microbiotas of sows in 18 farms. Panel A: Chao1 index; Panel B: Shannon index. The one-way analysis of variance (ANOVA) indicated that Chao1 and Shannon indices were of the indigenous microbiotas of sows in the farms (*p* < 0.05); Figure S2: Principal coordinate analysis (PCoA) based on weighted UniFrac distances generated from the fecal microbiotas of sows in 9 farms for group H (A), and 9 farms for group L (B). Ellipses were drawn to show non-statistical differences between the 9 farms in each group. The PERMANOVA analysis indicated that the compositions of the fecal microbiotas of sows differed between 9 farms in each group, as per the weighted UniFrac (*p* < 0.01). Figure S3: The weighted (A) and unweighted (B) UniFrac distance of fecal micobiotas in sows within the farm (intra-farm) and the distance against the sows different other farms in group H or L (inter-farm). The shaded, white and black boxes show the intra-farm distance, inter-farm distance (H group) and inter-farm distance (L group) respectively. The difference in UniFrac distance in each farm was analyzed by one-way ANOVA followed by Tukey post-hoc test (* *p* < 0.05, ** *p* < 0.01).

## References

[1] Britt J.H. (1986). Improving sow productivity through management during gestation, lactation and after weaning. J. Anim. Sci..

[2] Koketsu Y., Tani S., Iida R. (2017). Factors for improving reproductive performance of sows and herd productivity in commercial breeding herds. Porcine Health Manag..

[3] Quesnel H., Rodriguez-Martinez H., Vallet J.L., Ziecik A.J. (2009). Nutritional and lactational effects on follicular development in the pig. Control of Pig Reproduction VIII.

[4] Yun J., Valros A. (2015). Benefits of prepartum nest-building behaviour on parturition and lactation in sows—A review. Asian-Australas J. Anim. Sci..

[5] Luckey T.D. (1972). Introduction to intestinal microecology. Am. J. Clin. Nutr..

[6] Savage D.C. (1977). Microbial ecology of the gastrointestinal tract. Annu. Rev. Microbiol..

[7] Guarner F., Malagelada J.R. (2003). Gut flora in health and disease. Lancet.

[8] Sakata T. (2019). Pitfalls in short-chain fatty acid research: A methodological review. Anim. Sci. J..

[9] Louis P., Hold G.L., Flint H.J. (2014). The gut microbiota, bacterial metabolites and colorectal cancer. Nat. Rev. Microbiol..

[10] Tiwari U.P., Singh A.K., Jha R. (2019). Fermentation characteristics of resistant starch, arabinoxylan, and β-glucan and their effects on the gut microbial ecology of pigs: A review. Anim. Nutr..

[11] Tran T.H.T., Everaert N., Bindelle J. (2018). Review on the effects of potential prebiotics on controlling intestinal enteropathogens Salmonella and Escherichia coli in pig production. J. Anim. Physiol. Anim. Nutr..

[12] Fouhse J.M., Zijlstra R.T., Willing B.P. (2016). The role of gut microbiota in the health and disease of pigs. Anim. Front..

[13] Yang H., Huang X., Fang S., He M., Zhao Y., Wu Z., Yang M., Zhang Z., Chen C., Huang L. (2017). Unraveling the fecal microbiota and metagenomic functional capacity associated with feed efficiency in pigs. Front. Microbiol..

[14] Tan Z., Wang Y., Yang T., Ao H., Chen S., Xing K., Zhang F., Zhao X., Liu J., Wang C. (2018). Differences in gut microbiota composition in finishing Landrace pigs with low and high feed conversion ratios. Antonie Van Leeuwenhoek.

[15] McCormack U.M., Curião T., Buzoianu S.G., Prieto M.L., Ryan T., Varley P., Crispie F., Magowan E., Metzler-Zebeli B.U., Berry D. (2017). Exploring a possible link between the intestinal microbiota and feed efficiency in pigs. Appl Environ. Microbiol..

[16] Shao Y., Zhou J., Xiong X., Zou L., Kong X., Tan B., Yin Y. (2020). Differences in Gut Microbial and Serum Biochemical Indices Between Sows With Different Productive Capacities During Perinatal Period. Front. Microbiol..

[17] Xu K., Bai M., Liu H., Duan Y., Zhou X., Wu X., Liao P., Li T., Yin Y. (2020). Gut microbiota and blood metabolomics in weaning multiparous sows: Associations with oestrous. J. Anim. Physiol. Anim. Nutr..

[18] Koketsu Y. (2006). Sangyodobutsujuiryonihitsuyonaseisanshisutemunokangaekata. J. Japan Vet. Med. Assoc..

[19] Kure K. (2004). tonshashisetsu kanrikizai tonshakankyonadonikansurukadai (The Subjects about Institutions, Managed Equipments and Environment of Pig House). Jpn. J. Swine Sci..

[20] Yamane I. (2013). How to Handle Data in Epidemiological Research―An Example from Swine Benchmarking System. J. Vet. Epidemiol..

[21] Inoue R., Sakaue Y., Sawai C., Sawai T., Ozeki M., Romero-Pérez G.A., Tsukahara T. (2016). A preliminary investigation on the relationship between gut microbiota and gene expressions in peripheral mononuclear cells of infants with autism spectrum disorders. Biosci. Biotechnol. Biochem..

[22] Tsukahara T., Matsukawa N., Tomonaga S., Inoue R., Ushida K., Ochiai K. (2014). High-sensitivity detection of short-chain fatty acids in porcine ileal, cecal, portal and abdominal blood by gas chromatography-mass spectrometry. Anim. Sci. J..

[23] Morishima S., Aoi W., Kawamura A., Kawase T., Takagi T., Naito Y., Tsukahara T., Inoue R. (2020). Intensive, prolonged exercise seemingly causes gut dysbiosis in female endurance runners. J. Clin. Biochem. Nutr..

[24] Parks D.H., Tyson G.W., Hugenholtz P., Beiko R.G. (2014). STAMP: Statistical analysis of taxonomic and functional profiles. Bioinformatics.

[25] Qi K., Men X., Wu J., Xu Z. (2019). Rearing pattern alters porcine myofiber type, fat deposition, associated microbial communities and functional capacity. BMC Microbiol..

[26] He B., Bai Y., Jiang L., Wang W., Li T., Liu P., Tao S., Zhao J., Han D., Wang J. (2018). Effects of oat bran on nutrient digestibility, intestinal microbiota, and inflammatory responses in the hindgut of growing pigs. Int. J. Mol. Sci..

[27] Zhu J.J., Gao M.X., Song X.J., Zhao L., Li Y.W., Hao Z.H. (2018). Changes in bacterial diversity and composition in the faeces and colon of weaned piglets after feeding fermented soybean meal. J. Med. Microbiol..

[28] Onarman Umu Ö.C., Fauske A.K., Åkesson C.P., Pérez de Nanclares M., Sørby R., Press C.M., Øverland M., Sørum H. (2018). Gut microbiota profiling in Norwegian weaner pigs reveals potentially beneficial effects of a high-fiber rapeseed diet. PLoS ONE.

[29] Dehority B.A., Jung H.G., Buxton D.R., Hatfield R.D., Ralph J. (1993). Microbial ecology of cell wall fermentation. Forage Cell Wall Structure and Digestibility.

[30] Chassard C., Delmas E., Robert C., Lawson P.A., Bernalier-Donadille A. (2012). Ruminococcus champanellensis sp. nov., a cellulose-degrading bacterium from human gut microbiota. Int. J. Syst. Evol. Microbiol..

[31] Jiang X., Lu N., Xue Y., Liu S., Lei H., Tu W., Lu Y., Xia D. (2019). Crude fiber modulates the fecal microbiome and steroid hormones in pregnant Meishan sows. Gen. Comp. Endocrinol..

[32] Engelhardt W.V., Cummings J.H., Rombeau J.L., Sakata T. (1995). Absorption of short-chain fatty acids from the large intestine. Physiological and Clinical Aspects of Short-Chain Fatty Acids.

[33] Umesaki Y., Yajima T., Yokokuwa T., Mutai M. (1979). Effect of organic acid absorption or bicarbonate transport in rat colon. Pflügers Archiv..

[34] Shimotoyodome A., Meguro S., Hase T., Tokimitsu I., Sakata T. (2020). Short-chain fatty acids, but not lactate or succinate, stimulate mucus release in the rat colon. Comp. Biochem. Physiol..

[35] Smith P.M., Howitt M.R., Panikov N., Michaud M., Gallini C.A., Bohlooly M., Glickman J.N., Garrett W.S. (2013). The microbial metabolites, short-chain fatty acids, regulate colonic treg cell homeostasis. Science.

[36] Kimura-Todani T., Hata T., Miyata N., Takakura S., Yoshihara K., Zhang X.T., Asano Y., Altaisaikhan A., Tsukahara T., Sudo N. (2020). Dietary delivery of acetate to the colon using acylated starches as a carrier exerts anxiolytic effects in mice. Physiol. Behav..

[37] Segura M., Aragon V., Brockmeier S.L., Gebhart C., Greeff A.D., Kerdsin A., O’Dea M.A., Okura M., Saléry M. (2020). Update on Streptococcus suis Research and Prevention in the Era of Antimicrobial Restriction: 4th International Workshop on S. suis. Pathogens.

[38] Gottschalk M., Segura M., Xu J. (2017). Streptococcus suis infections in humans: The Chinese experience and the situation in North America. Anim. Health Res. Rev..

[39] Vigors S., O’Doherty J.V., Sweeney T. (2020). Colonic microbiome profiles for improved feed efficiency can be identified despite major effects of farm of origin and contemporary group in pigs. Animal.

[40] Oh J.K., Chae J.P., Pajarillo E.A.B., Kim S.H., Kwak M.J., Eun J.S., Chee S.W., Whang K., Kim A., Kang D.K. (2020). Association between the body weight of growing pigs and the functional capacity of their gut microbiota. Anim. Sci. J..

[41] Lin C., Wan J., Su Y., Zhu W. (2018). Effects of early intervention with maternal fecal microbiota and antibiotics on the gut microbiota and metabolite profiles of piglets. Metabolites.

[42] Yang Q., Huang X., Zhao S., Sun W., Yan Z., Wang P., Li S., Huang W., Zhang S., Liu L. (2017). Structure and function of the fecal microbiota in diarrheic neonatal piglets. Front. Microbiol..

[43] Modolo J.R., Margato L.F.F., Gottschalk A.F., Lopes C.A.D.M. (1999). Incidence of Campylobacter in pigs with and without diarrhea. Rev. Microbiol..

[44] De Rodas B., Youmans B.P., Danzeisen J.L., Tran H., Johnson T.J. (2018). Microbiome profiling of commercial pigs from farrow to finish. J. Anim. Sci..

[45] Jha R., Berrocoso J.F. (2016). Dietary fiber and protein fermentation in the intestine of swine and their interactive effects on gut health and on the environment: A review. Anim. Feed Sci. Technol..

[46] Isaacson R., Kim H.B. (2012). The intestinal microbiome of the pig. Anim. Health Res. Rev..

[47] Patil Y., Gooneratne R., Ju X.H. (2020). Interactions between host and gut microbiota in domestic pigs: A review. Gut Microbes.

[48] Kubasova T., Davidova-Gerzova L., Merlot E., Medvecky M., Polansky O., Gardan-Salmon D., Quesnel H., Rychlik I. (2017). Housing systems influence gut microbiota composition of sows but not of their piglets. PLoS ONE.

[49] Arruda A.G., Deblais L., Hale V., Pairis-Garcia M., Srivastava V., Kathayat D., Kumar A., Rajashekara G. (2019). Nasal and gut microbiota for sows of different health status within six commercial swine farms from one swine production system. BioRxiv.

