# Supplementary material for: Comparison of Productivity and Fecal Microbiotas of Sows in Commercial Farms
Source: Microorganisms. 2020 Sep 24;8(10):1469. doi: 10.3390/microorganisms8101469 (PMC7599717; doi:10.3390/microorganisms8101469)
Supplement: Supplementary file 1 [file microorganisms-08-01469-s001.pdf]

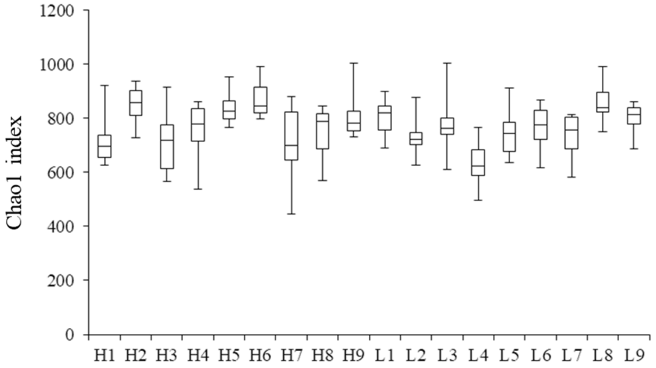

(A)

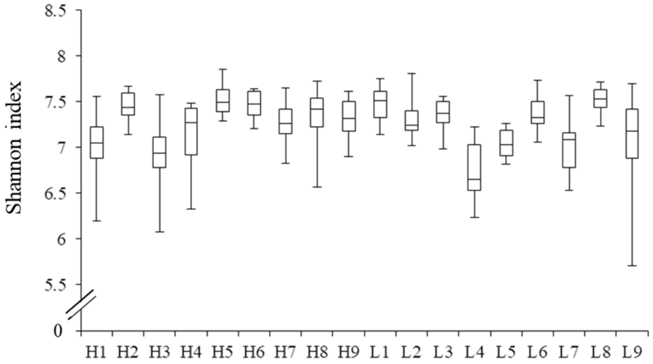

(B)

Supplementary Figure S1 Chao1 (**A**) and Shannon (**B**) indices of the fecal microbiotas of sows in 18 farms. Panel A: Chao1 index; Panel B: Shannon index. The one-way analysis of variance (ANOVA) indicated that Chao1 and Shannon indices were of the indigenous microbiotas of sows in the farms ($P < 0.05$).

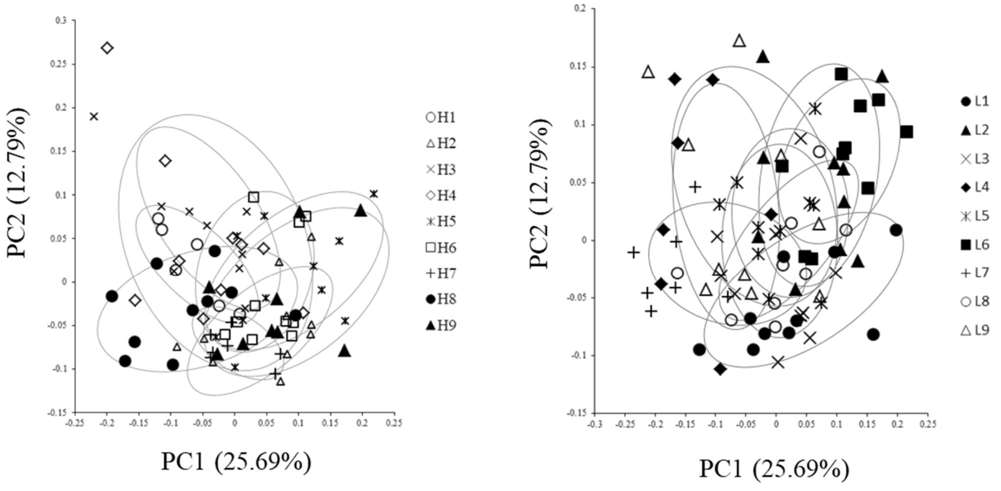

(A) (B)

Supplementary Figure S2 Principal coordinate analysis (PCoA) based on weighted UniFrac distances generated from the fecal microbiotas of sows in 9 farms for group H **(A),** and 9 farms for group L **(B).** Ellipses were drawn to show non-statistical differences between the 9 farms in each group. The PERMANOVA analysis indicated that the compositions of the gut microbiotas of sows differed between 9 farms in each group, as per the weighted UniFrac (P<0.01).

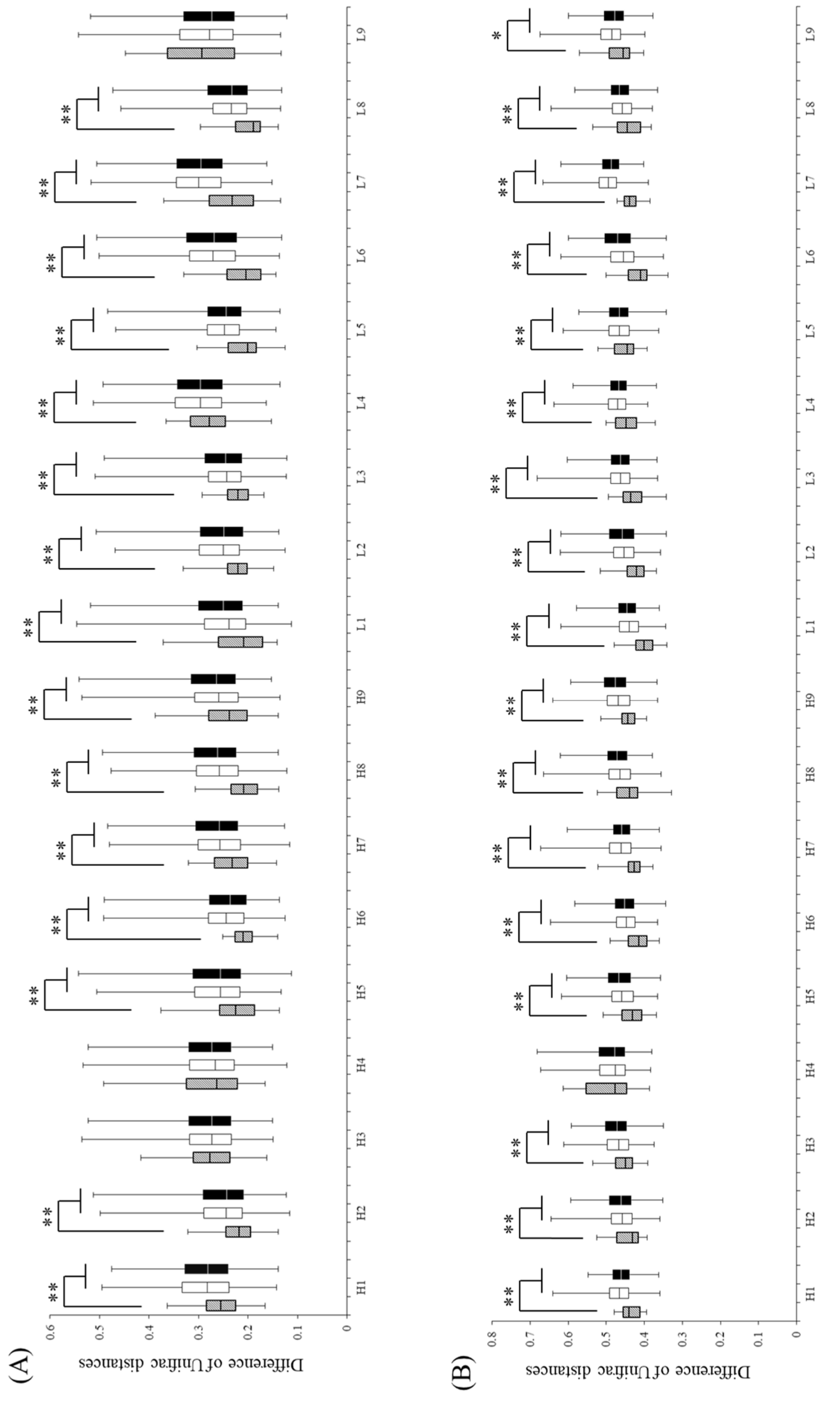

Supplementary Figure S3 The weighted (**A**) and unweighted (**B**) UniFrac distance of fecal micobiotas in sows within the farm (intra-farm) and the distance against the sows different other farms in group H or L (inter-farm). The shaded, white and black boxes show the intra-farm distance, inter-farm distance (H group) and inter-farm distance (L group) respectively. The difference in UniFrac distance in each farm was analyzed by one-way ANOVA followed by Tukey post-hoc test (*$P < 0.05$, **$P < 0.01$).
